# A multi-omics atlas of CAF subtypes reveals apCAF–M2 macrophage interactions driving immune resistance in glioma

**DOI:** 10.1371/journal.pone.0329801

**Published:** 2025-08-11

**Authors:** Yubo Ren, Dengfeng Lu, Fei Wang, Zixuan Wang, Jinfeng Li, Run Huang, Yue Lu, Aojie Duan, Renjie Shou, Jiangang Liu, Zhouqing Chen, Zhong Wang, Xiaoou Sun

**Affiliations:** 1 Department of Neurosurgery, The First Affiliated Hospital of Soochow University, Suzhou, Jiangsu Province, China; 2 Suzhou Medical College of Soochow University, Suzhou, Jiangsu Province, China; The University of Sheffield, UNITED KINGDOM OF GREAT BRITAIN AND NORTHERN IRELAND

## Abstract

Cancer-associated fibroblasts (CAFs) are a critical component of the glioma microenvi-ronment and play essential roles in tumor progression and resistance to immunotherapy. To comprehensively characterize CAF heterogeneity and their interactions with immune cells, we conducted an integrative multi-omics analysis incorporating single-cell and bulk RNA sequencing, spatial transcriptomics, and multiplex immunofluorescence. This approach identified nine distinct CAF subtypes with phenotypic and functional diversity, including tumor-like CAFs (tCAFs), myofibroblast-like CAFs (myCAFs), vascular CAFs (vCAFs), metabolic CAFs (meCAFs), proliferative CAFs (pCAFs), antigen-presenting CAFs (apCAFs), interferon-responsive CAFs (infCAFs), inflammatory CAFs (iCAFs), and a group of CAFs with unknown identity. Several subtypes were significantly associated with poor clinical outcomes. Notably, apCAFs engaged in extensive crosstalk with M2-polarized macrophages via TGF-β signaling pathways. Spatial transcriptomic pro-filing and immunofluorescence imaging revealed the co-localization of apCAFs and M2 macrophages at the tumor periphery, indicating the formation of an immunosuppressive niche. Moreover, AQP4 was identified as a specific marker of apCAFs, and its expression was significantly correlated with poor prognosis and resistance to immunotherapy. These findings offer a comprehensive atlas of CAF heterogeneity in glioma and highlight the therapeutic promise of targeting apCAF–M2 macrophage interactions or AQP4 to over-come immune resistance and improve clinical outcomes.

## Introduction

Gliomas are the most common primary central nervous system (CNS) tumors, accounting for 81% of malignant CNS tumors [1,2]. These tumors are classified into different grades based on their histological characteristics and malignancy levels. Among them, glioblastoma (GBM) is highly heterogeneous, and despite receiving standard treatment (the STUPP protocol, which includes surgery, radiotherapy, and chemotherapy), the prognosis remains poor, with a median overall survival of only 14.6 months [3,4]. In recent years, immunotherapy has rapidly advanced in cancer treatment, benefiting many patients with various malignancies. However, the clinical efficacy of immunotherapy in GBM patients remains disappointing [5]. This is largely attributed to the unique immunosuppressive microenvironment within GBM [6]. Therefore, elucidating the molecular mechanisms underlying tumor-induced immunosuppression and developing novel therapeutic strategies targeting these mechanisms are crucial for improving clinical treatment outcomes.

The tumor microenvironment (TME) of gliomas is a complex and dynamic system composed of various cellular and non-cellular components that interact with tumor cells, influencing tumor progression and therapeutic responses. Immune cells within the TME, such as myeloid-derived suppressor cells (MDSCs), glioma-associated macrophages/microglia (GAMs), and regulatory T cells (Tregs), contribute to immune evasion, promoting tumor progression, angiogenesis, invasion, and metastasis, thereby establishing an immunosuppressive TME [7–9]. In addition to immune cells, the TME includes the extracellular matrix, vasculature, and stromal cells, all of which play critical roles in supporting tumor growth and invasion [10–12]. Among all stromal components in the TME, cancer-associated fibroblasts (CAFs) are one of the most abundant and essential elements, playing a significant role in tumor progression and therapeutic resistance. CAFs are known to support tumor cell proliferation, remodel the extracellular matrix, promote angiogenesis, and mediate immunosuppression, thereby facilitating cancer progression [13,14].

One of the key roles of CAFs in the TME is their involvement in immune regulation. CAFs secrete cytokines and chemokines to recruit and modulate immune cells, contributing to the formation of an immunosuppressive environment that helps tumors evade immune recognition [15,16]. CAFs also play a crucial role in therapy resistance. They have been found to enhance cancer cell resistance to chemotherapy and radiotherapy. For instance, CAFs can secrete factors such as hepatocyte growth factor (HGF), activating signaling pathways like c-Met, which is associated with increased radioresistance in breast cancer [17]. Moreover, CAFs can induce epithelial-mesenchymal transition (EMT) in cancer cells, a process linked to increased invasiveness and therapy resistance [18]. Meanwhile, CAFs exhibit significant heterogeneity, reflecting their diverse origins and functions within the TME [19]. Different CAF subtypes have varying impacts on tumor growth and treatment responses, highlighting their potential as targets for precision therapy [20,21].

A series of studies have reported the critical roles of CAFs in tumor progression, immune evasion, and therapy resistance across various cancers [16,22–24]. However, a systematic analysis of CAFs in gliomas has yet to be reported, and their origins, subtypes, and functional characteristics remain unclear. Therefore, constructing a comprehensive atlas of glioma-associated CAFs is urgently needed. Such an atlas would not only enable a deeper understanding of their roles in glioma progression and immune resistance but also provide potential guidance for future precision therapeutic strategies targeting CAFs in gliomas.

## Materials and methods

### Data accessibility

The publicly available scRNA-seq datasets used in this study were obtained from various sources. The scRNA-seq data for glioma samples (GSE135045, GSE159416, GSE167960, GSE173278, GSE200984, GSE202371, GSE174554, GSE154795, GSE182109) were retrieved from the Gene Expression Omnibus (GEO) database (http://www.ncbi.nlm.nih.gov/GEO/). The scRNA-seq data for GBmap were obtained from the OSF database (https://osf.io/4q32e/, https://doi.org/10.17605/OSF.IO/4Q32E). The spatial transcriptomics data for glioma were derived from the 10X Genomics datasets (https://www.10xgenomics.com/cn) and GSE235672.

To investigate promising therapeutic targets in glioblastoma, this study established a discovery cohort consisting of 890 tumor samples of glioblastoma, each receiving transcriptome sequencing (RNA-seq) and with complete survival data. These subjects were drawn from seven distinct cohorts, including the Cancer Genome Atlas (TCGA, https://portal.gdc.cancer.gov), the Chinese Glioma Genome Atlas (CGGA, http://www.cgga.org.cn/index.jsp, CGGA693 and CGGA325), the Gene Expression Omnibus (GEO, https://www.ncbi.nlm.nih.gov/geo/, GSE121720 and GSE147352), the Glioma Longitudinal Analysis Series (GLASS, http://www.synapse.org/glass), and the Clinical Proteomic Tumor Analysis Consortium (CPTAC, https://pdc.cancer.gov/pdc/).

RNA-seq data and responses to immune checkpoint inhibitor (ICI) therapy were accessed from Braun, IMvigor, GSE91061, GSE78220, PRJNA482620 and PRJEB23709, focusing on anti-PD-1 treatment and anti-PD-L1 treatment.

### Single-cell data analysis

The scRNA-seq data were filtered using the R package Seurat (V.4.4.0) to retain cells that met the following criteria: the number of detected genes ranged between 500 and 7000, the mitochondrial gene proportion was below 30%, and the UMI count per cell was less than 30,000. Subsequently, a total of 240 single-cell samples and 26 single-cell datasets were batch-corrected and integrated using the harmony package in R. The integrated data were then standardized and normalized, and the FindVariableFeatures function was applied to identify the top 2000 highly variable genes. Principal component analysis (PCA) was performed on the HVGs, and the top 50 principal components were selected for subsequent cell clustering. Additionally, the FindNeighbors and FindClusters functions were used to identify cell subclusters, with a resolution of 0.8 set for all major cell types. Finally, uniform manifold approximation and projection (UMAP) was employed for visualization. The cell annotations were primarily referenced from previous studies [25–29].

### Functional enrichment analysis of cell subclusters

To investigate the biological functions associated with each cell subcluster, we performed functional enrichment analysis based on differentially expressed genes (DEGs) identified within each cluster. The DEGs were identified using the FindMarkers function in Seurat, with thresholds set at |log_2_FC| > 0.25 and an adjusted p-value < 0.05. For functional annotation, we conducted Gene Ontology (GO) enrichment analysis, including biological process (BP), cellular component (CC), and molecular function (MF) categories. These analyses were visualized using the clusterProfiler package in R.

### Cell development trajectory analysis

To explore potential cell differentiation pathways, we performed trajectory analysis using the Monocle2 package in R. First, a Monocle object was created using the newCellDataSet function. Next, genes were filtered based on recommended parameters suitable for downstream analysis. Dimensionality reduction was conducted using the reduceDimension function with the parameters reduction_method = “DDRTree” and max_components = 2. Cells were then ordered along a pseudotime sequence using the orderCells function, and the trajectory was visualized using the plot_cell_trajectory function. To illustrate the developmental progression of different CAF subtypes along pseudotime, we employed the plot_cell_trajectory function.

### Cell communication analysis

The CellChat package (version 1.6.1) [30] was used to infer potential communication between immune cells and different CAF subtypes within the tumor microenvironment. This cell-cell communication analysis was based on the ligand–receptor pairs database. First, we calculated the communication probability using the computeCommunProb function, followed by filtering out low-frequency interactions with the filterCommunication function. Second, the probability was assessed at the signaling pathway level using the computeCommunProbPathway function. Third, the comprehensive cell–cell communication network was identified using the aggregateNet function. Finally, specific signaling pathways were selected for visualization as heatmaps using the netVisual_heatmap function.

### Meta-analysis of immunotherapy response

Meta-analysis was performed utilizing “meta” package [31]. The gene expression data was initially log2-transformed, and subsequently converted to z-scores between patients in order to reduce heterogeneity between cohorts. Risk ratio (RR) for response meta-analysis and benjamini-adjusted hazard ratios (HR) calculated by univariate cox regression and 95% confidence intervals (CI) were calculated using the random-effect model, and the significance was determined by p < 0.05. We used Chi-squared and I2 statistics to assess statistical heterogeneity between studies, and if the p-value was considered significant, also we considered a value greater than 50% to be defined as substantial heterogeneity.

### Survival analysis

To assess the relationship between AQP4 gene expression and patient survival, we treated AQP4 expression levels as a variable and analyzed them in combination with glioma bulk RNA-seq data. The optimal expression cutoff for each gene was determined using the surv_cutpoint function in R, which stratified the data into high- and low-expression groups. Kaplan-Meier survival curves were generated using the survminer and survival R packages to visualize prognostic differences between the two groups. The difference in overall survival (OS) between groups was assessed using the log-rank test.

### ST analysis

The Seurat package (V.4.4.0) in R was used for pre-quality control (QC), clustering, and gene expression analysis of publicly available ST data. Principal component analysis (PCA) was applied for dimensionality reduction and clustering using the top 30 principal components (PCs). Next, we integrated the ST and scRNA-seq expression matrices using the CellTrek package in R to identify the spatial distribution of cell subpopulations. Additionally, spatial feature expression maps were generated using the “SpatialFeaturePlot” function in the Seurat package.

### Copy number variations detection

We used the R packages InferCNV [32] and copyKAT [33] to detect copy number variations (CNVs). In the InferCNV analysis, pDCs were used as the reference to estimate CNVs in other putative tumor cells. Only cells consistently classified as aneuploid by both InferCNV and copyKAT were considered malignant.

### Immunofluorescence (IF) staining

Since September 1, 2022, we have collected tumor tissue samples from glioma patients in the Department of Neurosurgery at the First Affiliated Hospital of Soochow University and established the “Gusu” glioma cohort. Subsequently, we randomly selected two GBM tissue sections from the Gusu cohort for immunofluorescence staining. The paraffin sections were first placed in a 70°C oven for 2–3 hours, followed by sequential deparaffinization in xylene (15 minutes), 100% ethanol (5 minutes), 95% ethanol (5 minutes), and 90% ethanol (5 minutes), and then rinsed with distilled water. Antigen retrieval was performed using citrate antigen retrieval powder (Ketu Biotech, Cat: G1201), diluted in double-distilled water to 1000 mL, with sections heated to 98°C for 10 minutes, then naturally cooled and washed three times with PBS (pH 7.4) for 5 minutes each on a decolorizing shaker. The sections were blocked with 10% goat serum at room temperature for 30 minutes, followed by overnight incubation with primary antibodies, including HLA-DRA (1:500, Proteintech, Cat No: 68543–1-Ig), TAGLN (1:300, Santa Cruz Biotechnology, Cat No: sc-53932), CD68 (1:500, Abcam, Cat No: AB303565), AQP4 (1:500, Santa Cruz Biotechnology, Cat No: sc-32739), and CD163 (1:500, Proteintech, Cat No: 16646–1-AP). The appropriate fluorescent secondary antibodies were added and incubated at room temperature for 1 hour, followed by DAPI staining, coverslipping, and microscopic observation.

### Ethics statement

This study was a retrospective analysis based on previously collected clinical data and archived tissue samples. The data were accessed for research purposes on 28 March 2025. This study adhered to all relevant ethical guidelines and was approved by the Ethics Committee of the First Affiliated Hospital of Soochow University (Ethics approval number: 2025247). During data collection, the authors did not have access to any personally identifiable information. All data were fully anony-mized prior to analysis to ensure patient confidentiality and comply with institutional and national ethical standards. Given the retrospective nature of the study and the use of de-identified data, the requirement for obtaining informed consent was waived by the ethics committee.

### Statistical analysis

Data manipulation, analysis, and visualization were performed using R software, version 4.4.0. The relationship between continuous variables was evaluated using Spearman’s rank correlation coefficients. The Wilcoxon rank sum test was used to compare continuous variables between different groups. Kaplan-Meier survival curves were generated using the survminer and survival R packages to visualize prognostic differences between the two groups. For univariate cox proportional hazards regression models, we leveraged the coxph function from the R package “survival”. To further explore the biological pathways active within tumor cells, we applied the ssGSEA algorithm available in the R package “GSVA”. Throughout our analysis, all statistical tests conducted were two-tailed, and a p-value threshold of less than 0.05 was used to denote statistical significance.

## Result

### Construction and analysis of the glioma CAFs atlas

**Fig 1:**
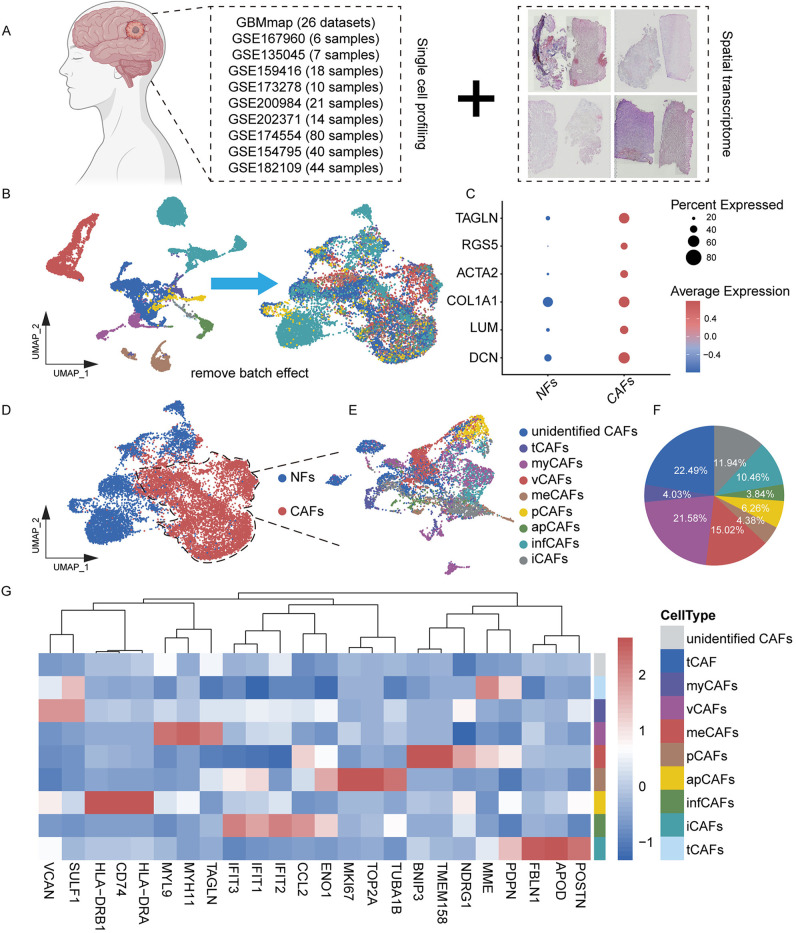
Comprehensive classification of fibroblasts through single-cell RNA sequencing analysis of glioma. ((A) Schematic diagram of data collection. (B) Comparison of single-cell data before and after batch effect removal. (C) In this study, fibroblasts were classified using specific biomarkers: DCN, LUM, and COL1A1 (for NFs), and ACTA2, RGS5, and TAGLN (for CAFs). (D) Based on these specific markers, fibroblasts were divided into NFs and CAFs. (E) CAFs were re-clustered using dimensionality reduction and annotated based on corresponding markers. (F) The pie chart shows the proportion of each CAFs subtype. (G) The dot plot illustrates the specific gene expression profiles of different CAFs subtypes.

Our study began with the construction of glioma CAFs atlas, derived from single-cell sequencing data of 240 samples across 26 datasets. Additionally, we utilized four publicly available spatial transcriptomics datasets to validate spatial localization (Fig 1A). A standard single-cell analysis pipeline was applied to each dataset and sample, including quality control, filtering, batch effect correction, dimensionality reduction, and clustering. Using specific biomarkers, we identified and extracted fibroblasts from each dataset.

Next, we aggregated the extracted fibroblasts, performed batch correction, dimensionality reduction, and unsupervised clustering, and identified 18 distinct cell clusters (S1A Fig). Based on specific biomarkers, we further classified fibroblasts into CAFs and normal fibroblasts (NFs) (Fig 1B). NFs were identified using established markers such as DCN, LUM, and COL1A1, while CAFs were characterized by biomarkers such as ACTA2, TAGLN, and CTHRC1 [25,34,35] (Fig 1C, D).

### Identification and characterization of biomarkers in the nine CAF subtypes

We extracted CAFs, reprocessed them through dimensionality reduction and integration, and identified nine distinct CAF subtypes: tumor-like CAFs (tCAFs), myofibroblasts-like CAFs (myCAFs), vascular CAFs (vCAFs), metabolic CAFs (meCAFs), proliferative CAFs (pCAFs), antigen-presenting CAFs (apCAFs), interferon-response CAFs (infCAFs), inflammatory CAFs (iCAFs) and unidentified CAFs (Fig 1E, F). These subtypes were classified based on the relative abundance and expression levels of specific biomarkers, with each subtype exhibiting a unique set of signature genes. tCAFs were primarily characterized by the expression of PDPN, MME, and TMEM158, while myCAFs expressed VCAN, SULF1, and POSTN [27,28,36]. vCAFs were identified by MYL9, MYH11, and TAGLN, and meCAFs were characterized by NDRG1, ENO1, and BNIP3 [26,29]. pCAFs predominantly expressed TUBA1B, MKI67, and TOP2A, whereas apCAFs were enriched in HLA-DRA, CD74, and HLA-DRB1 [27,37,38]. infCAFs exhibited high expression of IFIT1, IFIT2, and IFIT3, while iCAFs were defined by APOD, FBLN1, and CCL2 [38–40] (Fig 1G). Finally, CAFs lacking specific biomarkers were classified as unidentified CAFs.

**Fig 2:**
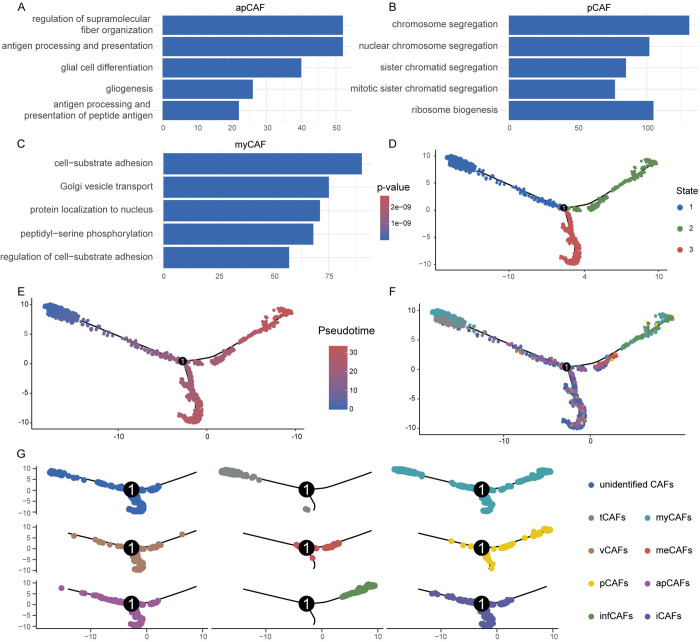
Functional enrichment and pseudotime analysis of CAFs subtypes. (A–(A–C) Functional enrichment results of apCAFs, pCAFs, and myCAFs. (D) Different differentiation stages of CAFs revealed by pseudotime analysis. (E) Pseudotime trajectory of CAFs generated using the DDRTree dimensionality reduction method. (F) The panel shows the distribution of different CAFs subtypes along the pseudotime trajectory. (G) Pseudotime differentiation trajectories of each CAF subtype.

Next, we performed functional enrichment analysis for each CAF subtype and found that the enrichment results were consistent with their expected biological roles (Fig 2A–C, S1B–F Fig). apCAFs were enriched in pathways related to antigen processing and presentation, as well as glial cell formation and differentiation. pCAFs were primarily associated with mitosis and cell proliferation, while myCAFs were enriched in pathways related to the extracellular matrix and collagen organization. We then conducted pseudotime trajectory analysis to explore the developmental relationships among the CAF subtypes. The analysis revealed that CAFs development progressed through three stages, where state 1 represented the early developmental stage, while state 2 and state 3 were considered mid-to-late developmental stages (Fig 2D, E). Unidentified CAFs were found at the earliest developmental stage, suggesting that they may serve as the origin of CAFs differentiation. As the tumor progressed, marker gene expression levels of myCAFs, tCAFs, vCAFs, meCAFs, pCAFs, apCAFs, and iCAFs gradually increased. Ultimately, vCAFs, apCAFs, and iCAFs converged into one developmental endpoint, while myCAFs, pCAFs, and infCAFs formed another terminal differentiation branch (Fig 2F, G). These findings highlight the distinct developmental heterogeneity among different CAF subtypes.

### Interaction analysis between CAF subtypes and immune cells

**Fig 3:**
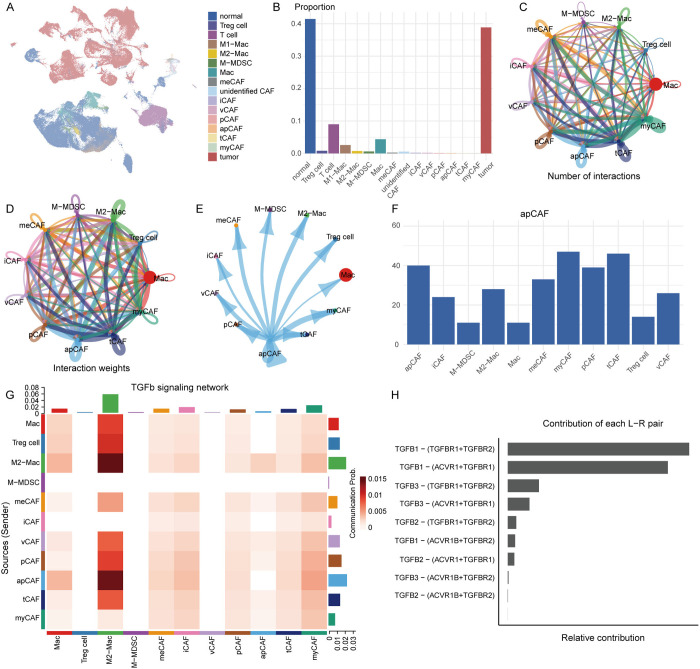
Exploring the interactions between CAFs subtypes and other cell types in the GSE182109 dataset. (A) (A) UMAP plot of single-cell data from the GSE182109 dataset. (B) Bar plot showing the proportion of each cell type. (C–D) Number of ligand-receptor pairs and communication strength between different cell populations. (E) Cell-cell communication network between apCAFs and other cell types. (F) Bar plot showing the interaction strength between apCAFs and other cells. (G) apCAFs exhibit the strongest interaction with M2 macrophages through the TGF-β signaling pathway. (H) Proportions of ligand-receptor pairs involved in the TGF-β signaling pathway.

In the GSE182109 glioma dataset, we explored the interaction frequency and strength between different CAF subtypes and other cell types. First, we performed dimensionality reduction, clustering, and cell annotation on the dataset (Fig 3A), which contained both normal tissue as well as CAF subtypes, tumor cells, and other immune cells (Fig 3B).

Next, we extracted CAF subtypes and immune cells for cell-cell interaction analysis, generating interaction networks (Fig 3C, D, S2 Fig). The results showed that apCAFs exhibited the strongest and most frequent interactions with immune cells, particularly M2 macrophages (Fig 3E, F). To further investigate the potential communication mechanisms between apCAFs and M2 macrophages, we performed cell-cell communication analysis and found that the TGF-β signaling pathway played a major role, where apCAFs acted as the ligand senders and M2 macrophages as the receptor cells, forming the most significant interaction (Fig 3H).

Further analysis revealed that within the TGF-β signaling pathway, the interaction between TGF-β1 and its receptors TGFBR1 and TGFBR2 was the most frequent (Fig 3G), suggesting that apCAFs may exert their effects on M2 macrophages through TGF-β1 binding to TGFBR1 and TGFBR2. Previous studies have shown that TGF-β1 is a key inducer of endothelial-to-mesenchymal transition (EndoMT) and is involved in the regulation of multiple cellular biological processes [41]. EndoMT is a special form of EMT that has been implicated in cancer progression and metastasis [42].Additionally, we found that among all CAF subtypes, aquaporin4 (AQP4) was specifically expressed in apCAFs, while other CAF subtypes did not express AQP4. This pattern was also confirmed in the GSE182109 dataset, where AQP4 expression was exclusively detected in apCAFs (S1G, H Fig).

### Risk profiling of CAF subtypes and the role of AQP4 in glioma immune resistance and prognosis

**Fig 4:**
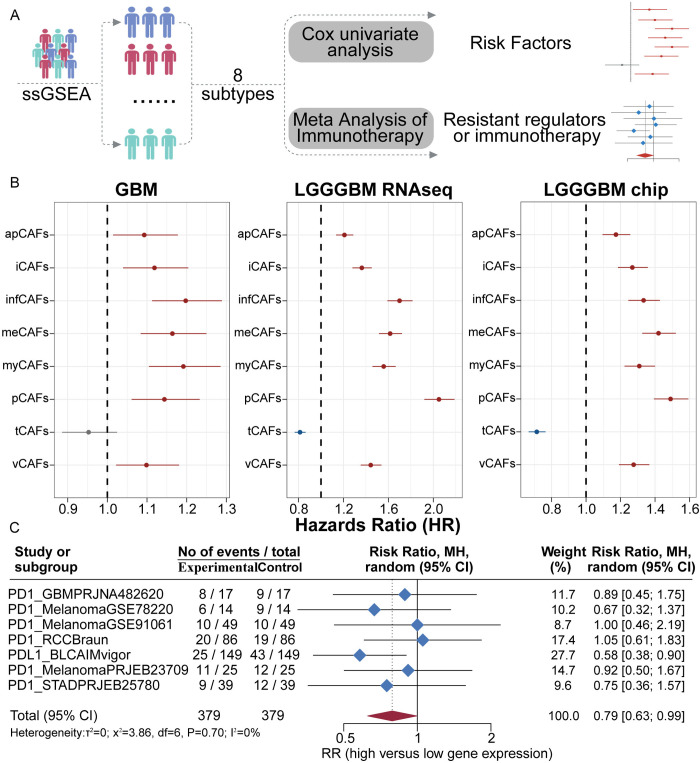
Meta-analysis evaluating the risk and immunotherapy response potential of CAFs subtypes. (A) Flowchart of the meta-analysis for identifying risk factors and assessing immunotherapy response. (B) Identification of risk-associated factors within each CAFs subtype. (C) Meta-analysis evaluating the association between apCAFs and tumor immunotherapy response.

Meanwhile, we conducted immune infiltration analysis using ssGSEA on eight immunotherapy cohorts and evaluated the risk and immune resistance of different CAF subtypes through Cox univariate analysis and meta-analysis of immunotherapy outcomes (Fig 4A). In both low-grade gliomas (LGG) and GBM, apCAFs, iCAFs, infCAFs, meCAFs, myCAFs, pCAFs, and vCAFs were identified as high-risk factors, whereas tCAFs were not considered a risk factor (Fig 4B). Moreover, in the context of immunotherapy, we found that the high apCAFs score group exhibited stronger immune resistance compared to the low score group (Fig 4C).

**Fig 5:**
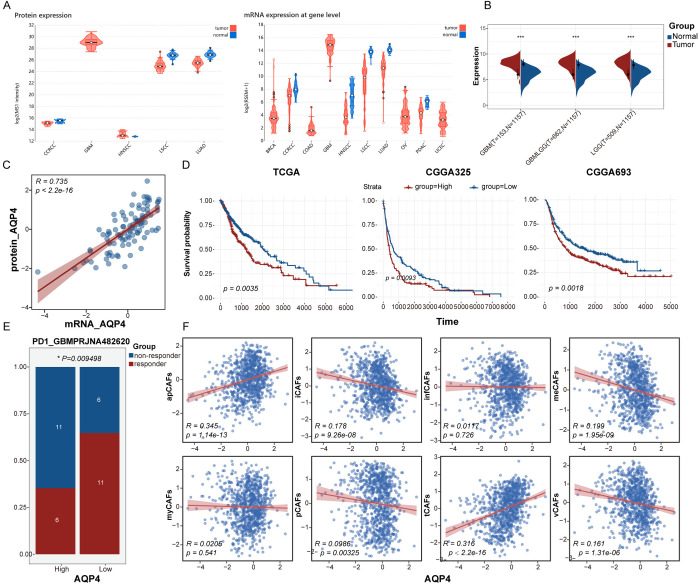
Analysis of AQP4 at the bulk RNA-seq level. (A) (A) mRNA and protein expression levels of AQP4 across various cancer types. (B) Expression levels of AQP4 in low-grade and high-grade gliomas. (C) Correlation analysis between AQP4 mRNA and protein expression. (D) Impact of AQP4 expression levels on patient prognosis. (E) Influence of AQP4 expression levels on immunotherapy response. (F) Correlation analysis between AQP4 and different CAF subtypes.

In bulk RNA sequencing and proteomic analyses across various cancer types, AQP4 consistently shows higher expression at both the mRNA and protein levels in glioma tissues compared to normal tissues (Fig 5A). In both LGG and high-grade gliomas (HGG), AQP4 expression remained significantly higher in tumor tissues compared to normal tissues, and AQP4 mRNA levels correlated strongly with its protein expression (Fig 5B, C). TCGA data analysis revealed that patients in the AQP4 high-expression group had shorter survival than those in the low-expression group, a finding that was further validated in both GEO and CGGA datasets (Fig 5D). In PD-1-targeted immunotherapy, the AQP4 high-expression group exhibited stronger immune tolerance compared to the low-expression group (Fig 5E). Additionally, we investigated the correlation between AQP4 expression and CAF subtypes. The results showed that AQP4 was significantly associated with apCAFs, whereas no clear correlation was observed with other CAF subtypes (Fig 5F).

### Spatial co-localization of apCAFs and M2 macrophages in the tumor microenvironment

**Fig 6:**
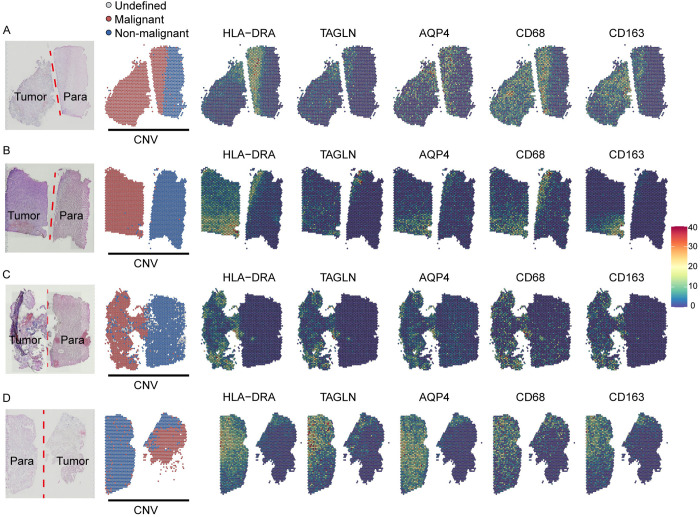
Spatial relationship between apCAFs and M2 macrophages within the tumor microenvironment. (A–D) Spatial transcriptomics analysis reveals that marker genes of apCAFs and M2 macrophages are localized at the tumor margins.

**Fig 7:**
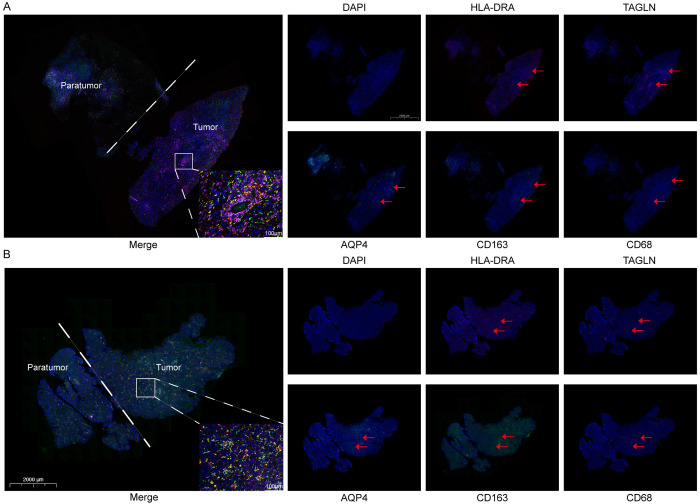
Multiplex immunofluorescence staining of tumor and peritumoral tissues from patients. (A-B) Immunofluorescence staining of glioma patient tissue sections. DAPI (blue), HLA-DRA (red), TAGLN (pink), AQP4 (cyan), CD163 (green), and CD68 (yellow) were visualized in both individual channels and merged composite images.

To investigate the spatial relationship between apCAFs and M2 macrophages, we analyzed four publicly available spatial transcriptomic datasets. We first distinguished malignant from non-malignant cells through CNV analysis. Spatial mapping revealed a notable overlap between the specific marker genes of apCAFs and those of M2 macrophages (Fig 6A–D). Both cell types were predominantly localized at the tumor margin, with minimal accumulation observed within the tumor core. Furthermore, the apCAF marker AQP4 exhibited spatial co-localization with M2 macrophage markers in the same regions (Fig 6A–D). Consistently, spatial co-localization scoring revealed a certain degree of spatial proximity between the two cell types in the peritumoral region (S3A–D Fig). This spatial pattern was further confirmed by multiplex immunofluorescence staining of tumor and peritumoral tissues from patient samples, which showed consistent enrichment of apCAFs and M2 macrophages at the tumor edge (S3E, F Fig). These findings suggest that apCAFs and M2 macrophages may co-localize at the invasive front, potentially forming an immunosuppressive barrier that contributes to tumor immune evasion (Fig 7A, B).

## Discussion

In our study, we conducted a comprehensive analysis using publicly available scRNA-seq and bulk RNA sequencing datasets, along with ST data from glioma patients. This study primarily aimed to construct a comprehensive atlas of glioma-associated CAFs, investigate their classification and characterization within gliomas, and provide valuable insights into their properties at the single-cell level.

The heterogeneity of CAFs plays a crucial regulatory role in the immune microenvironment. In this study, we systematically characterized the heterogeneity and features of glioma-associated CAFs and identified eight distinct subtypes: tCAFs, myCAFs, vCAFs, meCAFs, pCAFs, apCAFs, infCAFs, and iCAFs. Among them, apCAFs were characterized by the expression of HLA-DRA, CD74, and HLA-DRB1, which are associated with antigen processing and presentation, while myCAFs expressed VCAN, SULF1, and POSTN, indicating their role in collagen synthesis and extracellular matrix remodeling. vCAFs were identified by MYL9, MYH11, and TAGLN, suggesting their involvement in vascularization and vascular smooth muscle regulation within the TME. In bulk RNA-seq analysis, apCAFs, iCAFs, infCAFs, meCAFs, myCAFs, pCAFs, and vCAFs were all associated with poor prognosis in glioma. Additionally, evidence suggests that M2 macrophages play a critical role in glioma growth, angiogenesis, tumor infiltration, and therapeutic resistance [43,44]. Through cell-cell communication analysis, we found that apCAFs exhibited the strongest interaction with M2 macrophages, likely regulating them via the TGF-β signaling pathway. Furthermore, ST data revealed that apCAFs and M2 macrophages were primarily co-localized at the tumor periphery, suggesting a potential role in immune evasion, a finding further validated by immunofluorescence staining. Given that TGF-β is a key player in tumor invasion and metastasis [45], and that glioma-infiltrating macrophages and microglia can be recruited and induced into immunosuppressive phenotypes by TGF-β [46], targeting the TGF-β pathway with inhibitors or monoclonal antibodies may provide a promising therapeutic strategy for gliomas.

Additionally, we found that among the CAF subtypes, AQP4 was specifically enriched in apCAFs. Studies have shown that increased AQP4 expression is closely associated with poor tumor prognosis, and the immune evasion capability is stronger in the AQP4 high-expression group compared to the low-expression group. AQP4 is one of the major aquaporin (AQP) molecules in the central nervous system, regulating cerebrospinal fluid (CSF) flow, and contributing to cytotoxic and vasogenic edema [47]. A previous study found that macrophages in the high AQP4 expression group tend to polarize toward the M2 macrophage phenotype, whereas macrophages in the low AQP4 expression group did not show a clear polarization trend [48]. Another study demonstrated that targeted inhibition of AQP4 alleviated radiation-induced lung injury and suppressed M2 macrophage activation [49]; however, similar studies in gliomas remain limited. Given its potential role in glioma progression and the tumor immunosuppressive microenvironment, further investigation into the mechanisms of AQP4 may provide novel therapeutic insights for glioma treatment.

Our study also has several limitations. For instance, the glioma single-cell datasets we used predominantly consisted of GBM, with relatively fewer LGG samples included. Additionally, although we identified that apCAFs regulate M2 macrophages through the TGF-β pathway, the underlying biological mechanisms remain to be further investigated. Furthermore, the specific biological mechanisms by which AQP4 influences glioma prognosis are still unknown, and its functional role within apCAFs requires further exploration.

## Conclusion

In conclusion, our study comprehensively analyzed the heterogeneity of glioma-associated CAFs and systematically constructed their single-cell atlas. This research provides a new perspective on the classification of glioma CAFs and their role within the tumor microenvironment, offering potential novel targets for glioma immunotherapy.

## Supporting information

S1 Fig Clustering and characterization analysis of CAFs subtypes. (A) Dimensionality reduction and clustering plot of CAFs subtypes. (B–F) Functional enrichment results for tCAFs, vCAFs, iCAFs, infCAFs, and meCAFs, respectively. (G) Expression level of AQP4 in the GSE182109 dataset. (H) Expression of AQP4 across different CAFs subtypes.
(TIF)

S2 Fig Cell–cell communication analysis of different cell types in the GSE182109 dataset. (TIF)

S3 Fig Spatial analysis of apCAFs and M2 macrophages based on spatial transcriptomics and immunofluorescence. (A-D) Co-localization score analysis of apCAFs and M2 macrophages. (E-F) Immunofluorescence-based spatial proximity analysis of apCAFs and M2 macrophages.
(TIF)

S4 Data Immunofluorescence images and spatial transcriptomics data. (ZIP)

## Abbreviations

CAFs: cancer-associated fibroblasts
TME: tumor microenvironment
CNS: central nervous system
GBM: glioblastoma
MDSCs: myeloid-derived suppressor cells
GAMs: glioma-associated macrophages/microglia
Tregs: regulatory T cells
HGF: hepatocyte growth factor
EMT: epithelial-mesenchymal transition
tCAFs: tumor-like CAFs
myCAFs: myofibroblasts-like CAFs
vCAFs: vascular CAFs
meCAFs: metabolic CAFs
pCAFs: proliferative CAFs
apCAFs: antigen-presenting CAFs
infCAFs: interferon-response CAFs
iCAFs: inflammatory CAFs
EndoMT: endothelial-to-mesenchymal transition
ST: spatial transcriptomics
LGG: low-grade gliomas
AQP4: aquaporin4
HGG: high-grade gliomas
CSF: cerebrospinal fluid
AQP: aquaporin
GEO: Gene Expression Omnibus
RNA-seq: transcriptome sequencing
ICI: immune checkpoint inhibitor
PCA: Principal component analysis
UMAP: uniform manifold approximation and projection
DEGs: differentially expressed genes
GO: Gene Ontology
BP: biological process
CC: cellular component
MF: molecular function
RR: Risk ratio
HR: hazard ratios
CI: confidence intervals
OS: overall survival
QC: pre-quality control
PCs: principal components
IF: Immunofluorescence
