# Supplementary figures and images for: A multi-omics atlas of CAF subtypes reveals apCAF–M2 macrophage interactions driving immune resistance in glioma
Source: PLoS One. 2025 Aug 11;20(8):e0329801. doi: 10.1371/journal.pone.0329801 (PMC12338775; doi:10.1371/journal.pone.0329801)

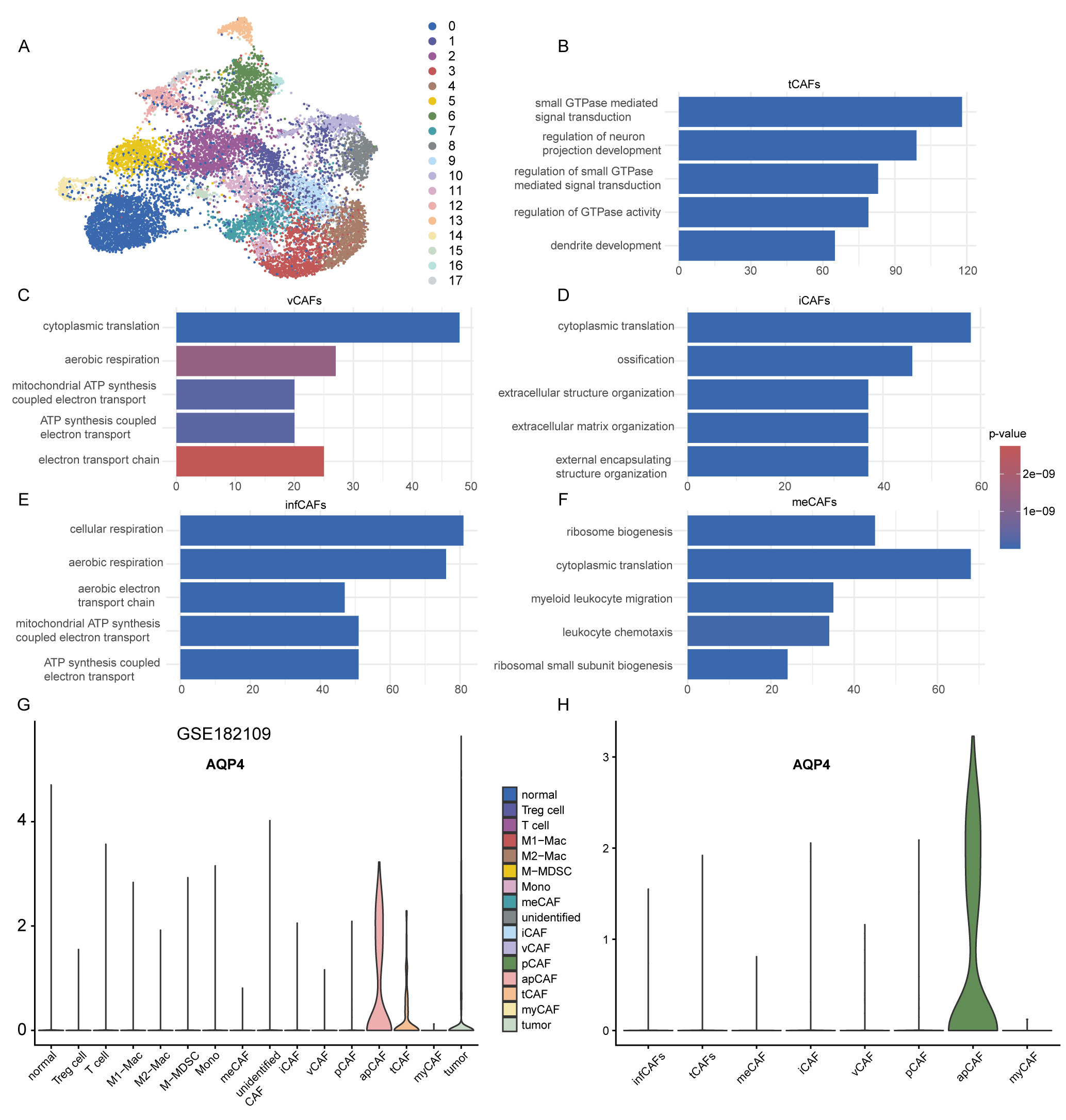

Supplement: S1 Fig — (A) Dimensionality reduction and clustering plot of CAFs subtypes. (B–F) Functional enrichment results for tCAFs, vCAFs, iCAFs, infCAFs, and meCAFs, respectively. (G) Expression level of AQP4 in the GSE182109 dataset. (H) Expression of AQP4 across different CAFs subtypes. (TIF) [file pone.0329801.s001.tif]

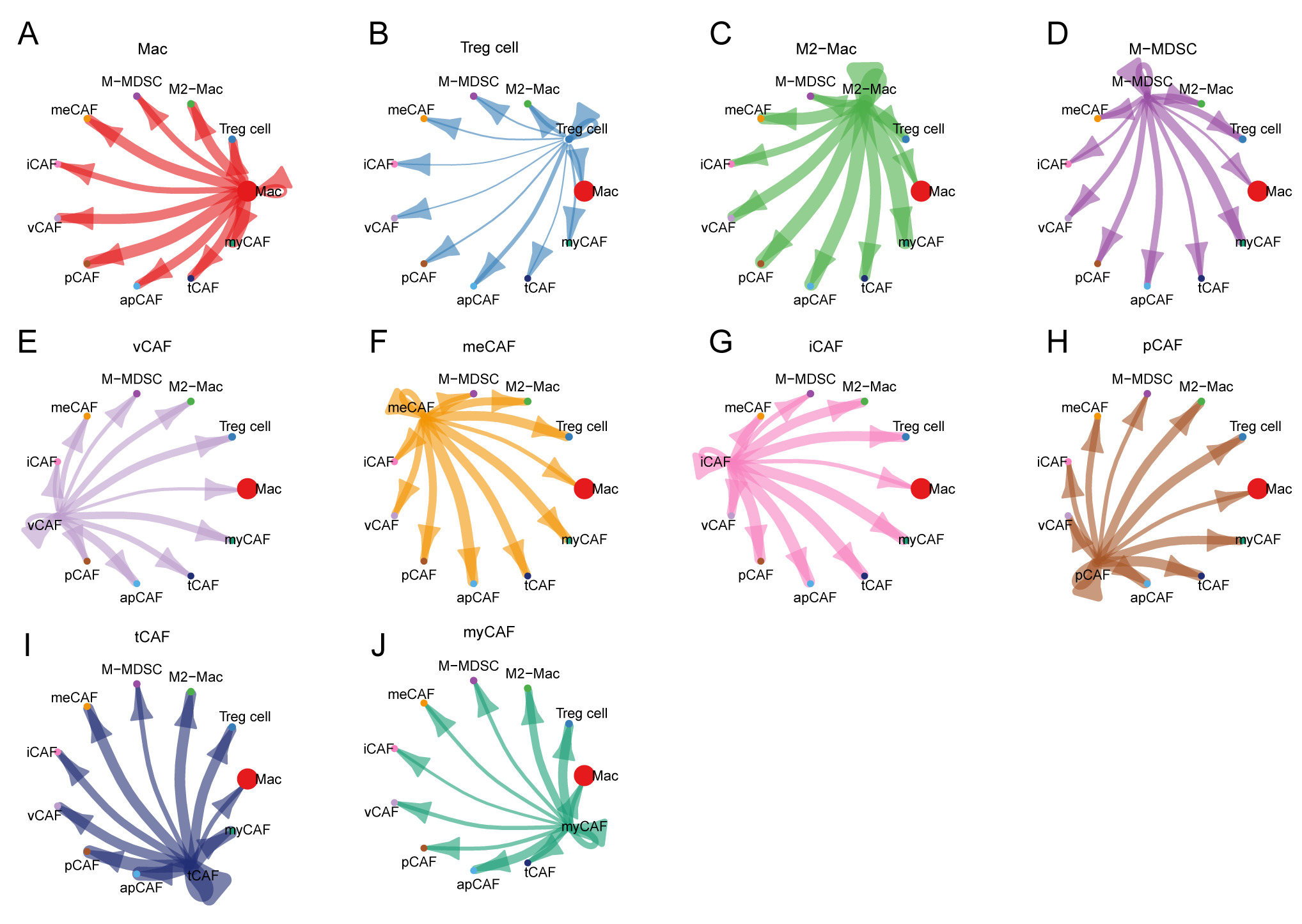

Supplement: S2 Fig — (TIF) [file pone.0329801.s002.tif]

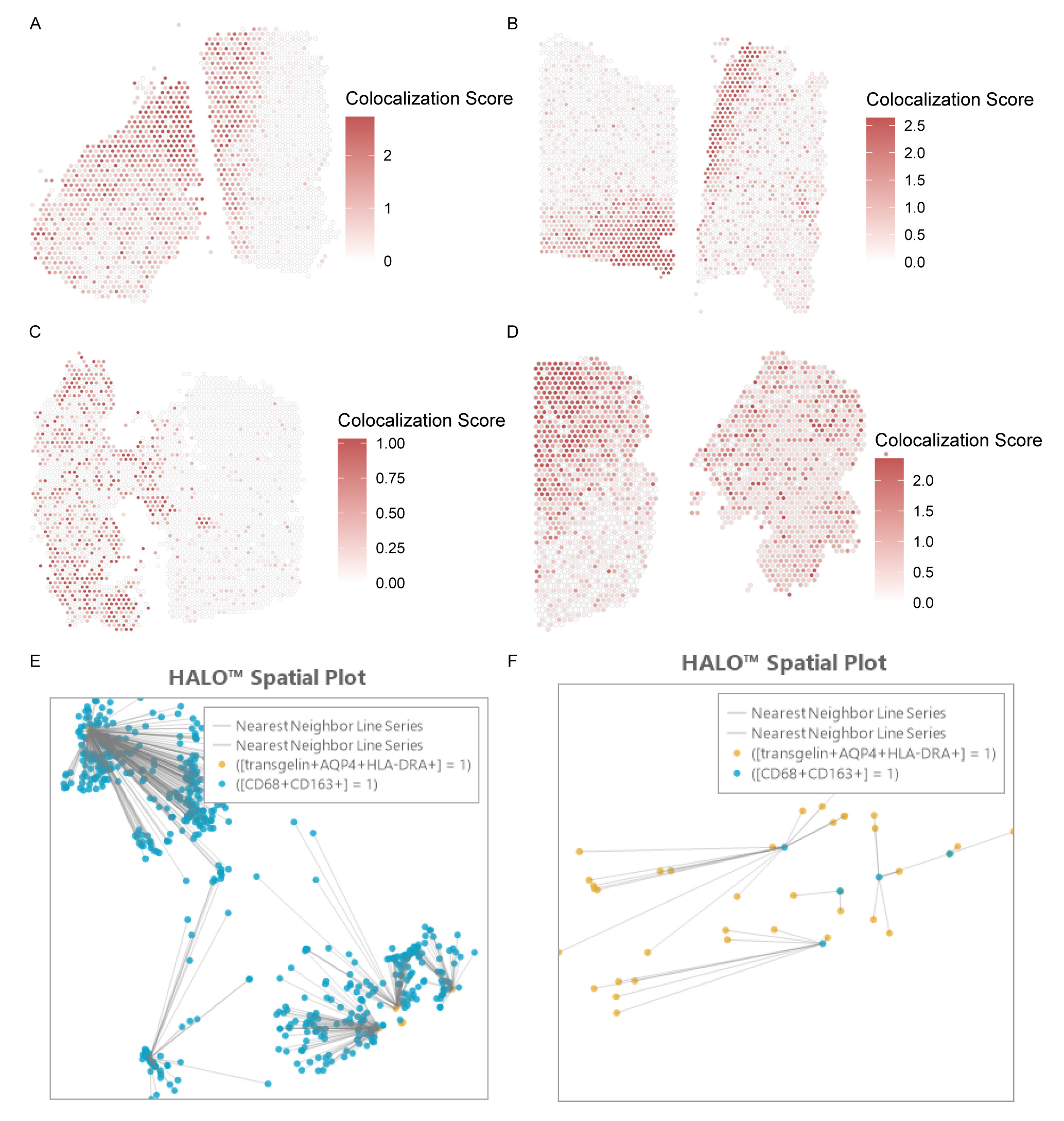

Supplement: S3 Fig — (A-D) Co-localization score analysis of apCAFs and M2 macrophages. (E-F) Immunofluorescence-based spatial proximity analysis of apCAFs and M2 macrophages. (TIF) [file pone.0329801.s003.tif]
