# Supplementary material for: A multi-omics atlas of CAF subtypes reveals apCAF–M2 macrophage interactions driving immune resistance in glioma
Source: PLoS One. 2025 Aug 11;20(8):e0329801. doi: 10.1371/journal.pone.0329801 (PMC12338775; doi:10.1371/journal.pone.0329801)
Supplement: Attachment — Submitted filename: Response to Reviewers.docx [file pone.0329801.s005.docx]

**Response to Reviewers**

**Reviewer #1:**

**1. When analyzing different data, was the batch effect removed?**

Thank you for your constructive suggestions. To correct for batch effects across datasets, we used the Harmony package. The integration results are presented in Figure 1B.

**2. What is the marker gene set for CAFs? Where was it obtained from?**

Thank you for your suggestion. We used ACTA2, TAGLN, and CTHRC1 as marker genes for cancer-associated fibroblasts (CAFs), and DCN, LUM, and COL1A1 as markers for normal fibroblasts. The marker genes for CAFs were obtained from the following studies (PMID: 37463917, 33622705, and 32434947), which have been cited in the main text.

**3. Why were only two patients selected for the immunofluorescence staining experiment? Are there specific values for spatial distance to illustrate the research conclusions? It is recommended to use HALO spatial coordinate analysis software to define the distance between different cell types.**

Thank you for your constructive suggestions. At present, the number of samples containing both tumor tissue and adjacent normal regions is limited. We plan to collect additional samples in future studies to further validate this observation. In response to your suggestion, we performed HALO spatial coordinate analysis, which revealed that apCAFs and M2 macrophages are spatially proximal at the tumor margin. The results of this analysis have been included in the supplementary materials (Newly Added Supplementary Figure 3).

**4. How is the spatial co-localization of AQP4 and M2 macrophage markers in the same area in ST defined? Was the spatial co-localization score used?**

Thank you for your suggestion. While our initial assessment of AQP4 and M2 macrophage co-localization was based on overlapping expression patterns, we have now incorporated a quantitative spatial co-localization score analysis, as recommended. These new results, obtained through normalized expression products and kNN-based neighborhood analysis, further support our original conclusion and have been included in Supplementary Figure 3.

**5. What is the expression pattern of AQP4 in immunofluorescence staining?**

Thank you for your suggestion. Immunofluorescence results show that the gene AQP4 is primarily expressed in apCAFs.

**Reviewer #2: Ren et al. analyzed publicly available scRNA-seq and bulk RNA sequencing datasets, as well as ST data from glioma patients. They investigated nine distinct CAF subtypes and mostly focused on apCAFs. Their data shows that apCAF2 is co-localized with M2 macrophages and there is a crosstalk between them via the TGF-b pathway. Patients with high apCAFs scores exhibited stronger immune resistance than patients with low apCAFs scores. Furthermore, they found that AQP4 is a specific marker for apCAFs and high expression of AQP4 in the tumor microenvironment indicated the immune resistance as well. Overall, the manuscript is well-written and presents interesting data.**

We are truly grateful for your thoughtful and constructive review of our manuscript. We greatly appreciate your insightful comments and your positive evaluation of our study.
