# Supplementary material for: A multi-omics atlas of CAF subtypes reveals apCAF–M2 macrophage interactions driving immune resistance in glioma
Source: PLoS One. 2025 Aug 11;20(8):e0329801. doi: 10.1371/journal.pone.0329801 (PMC12338775; doi:10.1371/journal.pone.0329801)
Supplement: Peer review history

# Peer review: Decision Letter 0

**DOI:** 10.1371/journal.pone.0329801.r001

**Authors:** Zeyneb Kurt

27 Jun 2025

PONE-D-25-29408A multi-omics atlas of CAF subtypes reveals apCAF–M2 macrophage interactions driving immune resistance in gliomaPLOS ONE

Dear Dr. Sun,

Thank you for submitting your manuscript to PLOS ONE. After careful consideration, we feel that it has merit but does not fully meet PLOS ONE’s publication criteria as it currently stands. Therefore, we invite you to submit a revised version of the manuscript that addresses the points raised during the review process.

Please submit your revised manuscript by Aug 11 2025 11:59PM. If you will need more time than this to complete your revisions, please reply to this message or contact the journal office at plosone@plos.org. When you're ready to submit your revision, log on to https://www.editorialmanager.com/pone/ and select the 'Submissions Needing Revision' folder to locate your manuscript file.

Please include the following items when submitting your revised manuscript:

- A rebuttal letter that responds to each point raised by the academic editor and reviewer(s). You should upload this letter as a separate file labeled 'Response to Reviewers'.
- A marked-up copy of your manuscript that highlights changes made to the original version. You should upload this as a separate file labeled 'Revised Manuscript with Track Changes'.
- An unmarked version of your revised paper without tracked changes. You should upload this as a separate file labeled 'Manuscript'.

If you would like to make changes to your financial disclosure, please include your updated statement in your cover letter. Guidelines for resubmitting your figure files are available below the reviewer comments at the end of this letter.

If applicable, we recommend that you deposit your laboratory protocols in protocols.io to enhance the reproducibility of your results. Protocols.io assigns your protocol its own identifier (DOI) so that it can be cited independently in the future. For instructions see: https://journals.plos.org/plosone/s/submission-guidelines#loc-laboratory-protocols. Additionally, PLOS ONE offers an option for publishing peer-reviewed Lab Protocol articles, which describe protocols hosted on protocols.io. Read more information on sharing protocols at https://plos.org/protocols?utm_medium=editorial-email&utm_source=authorletters&utm_campaign=protocols.

We look forward to receiving your revised manuscript.

Kind regards,

Zeyneb Kurt, PhD

Academic Editor

PLOS ONE

Journal Requirements:

When submitting your revision, we need you to address these additional requirements.

1. Please ensure that your manuscript meets PLOS ONE's style requirements, including those for file naming. The PLOS ONE style templates can be found at https://journals.plos.org/plosone/s/file?id=wjVg/PLOSOne_formatting_sample_main_body.pdf and https://journals.plos.org/plosone/s/file?id=ba62/PLOSOne_formatting_sample_title_authors_affiliations.pdf

2. Please note that PLOS One has specific guidelines on code sharing for submissions in which author-generated code underpins the findings in the manuscript. In these cases, we expect all author-generated code to be made available without restrictions upon publication of the work. Please review our guidelines at https://journals.plos.org/plosone/s/materials-and-software-sharing#loc-sharing-code and ensure that your code is shared in a way that follows best practice and facilitates reproducibility and reuse.

3. Please amend your list of authors on the manuscript to ensure that each author is linked to an affiliation. Authors’ affiliations should reflect the institution where the work was done (if authors moved subsequently, you can also list the new affiliation stating “current affiliation:….” as necessary).

4. Please include captions for your Supporting Information files at the end of your manuscript, and update any in-text citations to match accordingly. Please see our Supporting Information guidelines for more information: http://journals.plos.org/plosone/s/supporting-information.

5. Please review your reference list to ensure that it is complete and correct. If you have cited papers that have been retracted, please include the rationale for doing so in the manuscript text, or remove these references and replace them with relevant current references. Any changes to the reference list should be mentioned in the rebuttal letter that accompanies your revised manuscript. If you need to cite a retracted article, indicate the article’s retracted status in the References list and also include a citation and full reference for the retraction notice.

[Note: HTML markup is below. Please do not edit.]

Reviewers' comments:

Reviewer's Responses to Questions

**Comments to the Author**

1. Is the manuscript technically sound, and do the data support the conclusions?

The manuscript must describe a technically sound piece of scientific research with data that supports the conclusions. Experiments must have been conducted rigorously, with appropriate controls, replication, and sample sizes. The conclusions must be drawn appropriately based on the data presented. 

Reviewer #1: Yes

Reviewer #2: Yes

**********

2. Has the statistical analysis been performed appropriately and rigorously? 

Reviewer #1: Yes

Reviewer #2: Yes

**********

3. Have the authors made all data underlying the findings in their manuscript fully available?

The PLOS Data policy requires authors to make all data underlying the findings described in their manuscript fully available without restriction, with rare exception (please refer to the Data Availability Statement in the manuscript PDF file). The data should be provided as part of the manuscript or its supporting information, or deposited to a public repository. For example, in addition to summary statistics, the data points behind means, medians and variance measures should be available. If there are restrictions on publicly sharing data—e.g. participant privacy or use of data from a third party—those must be specified.

Reviewer #1: Yes

Reviewer #2: Yes

**********

4. Is the manuscript presented in an intelligible fashion and written in standard English?

PLOS ONE does not copyedit accepted manuscripts, so the language in submitted articles must be clear, correct, and unambiguous. Any typographical or grammatical errors should be corrected at revision, so please note any specific errors here.

Reviewer #1: Yes

Reviewer #2: Yes

**********

5. Review Comments to the Author

Please use the space provided to explain your answers to the questions above. You may also include additional comments for the author, including concerns about dual publication, research ethics, or publication ethics. (Please upload your review as an attachment if it exceeds 20,000 characters)

Reviewer #1: This study uses a combination of single-cell transcriptome, spatial transcriptome, bulk RNA-seq, and multiple immunofluorescence staining data to systematically reveal the heterogeneity of cancer-associated fibroblasts (CAFs) in glioma and its association with immune escape.

However, some questions need to be addressed

1. When analyzing different data, was the batch effect removed?

2. What is the marker gene set for CAFs? Where was it obtained from?

3. Why were only two patients selected for the immunofluorescence staining experiment? Are there specific values ​​for spatial distance to illustrate the research conclusions? It is recommended to use HALO spatial coordinate analysis software to define the distance between different cell types.

4. How is the spatial co-localization of AQP4 and M2 macrophage markers in the same area in ST defined? Was the spatial co-localization score used?

5. What is the expression pattern of AQP4 in immunofluorescence staining?

Reviewer #2: Ren et al. analyzed publicly available scRNA-seq and bulk RNA sequencing datasets, as well as ST data from glioma patients. They investigated nine distinct CAF subtypes and mostly focused on apCAFs. Their data shows that apCAF2 is co-localized with M2 macrophages and there is a crosstalk between them via the TGF-b pathway. Patients with high apCAFs scores exhibited stronger immune resistance than patients with low apCAFs scores. Furthermore, they found that AQP4 is a specific marker for apCAFs and high expression of AQP4 in the tumor microenvironment indicated the immune resistance as well. Overall, the manuscript is well-written and presents interesting data.

**********

6. PLOS authors have the option to publish the peer review history of their article (what does this mean?). If published, this will include your full peer review and any attached files.

If you choose “no”, your identity will remain anonymous but your review may still be made public.

**Do you want your identity to be public for this peer review?** For information about this choice, including consent withdrawal, please see our Privacy Policy.

Reviewer #1: No

Reviewer #2: **Yes: **Ece Tavukcuoglu

**********

[NOTE: If reviewer comments were submitted as an attachment file, they will be attached to this email and accessible via the submission site. Please log into your account, locate the manuscript record, and check for the action link "View Attachments". If this link does not appear, there are no attachment files.]

While revising your submission, please upload your figure files to the Preflight Analysis and Conversion Engine (PACE) digital diagnostic tool, https://pacev2.apexcovantage.com/. PACE helps ensure that figures meet PLOS requirements. To use PACE, you must first register as a user. Registration is free. Then, login and navigate to the UPLOAD tab, where you will find detailed instructions on how to use the tool. If you encounter any issues or have any questions when using PACE, please email PLOS at figures@plos.org. Please note that Supporting Information files do not need this step.

# Author comment: Author response to Decision Letter 1

**DOI:** 10.1371/journal.pone.0329801.r002

18 Jul 2025

Reviewer #1:

1. When analyzing different data, was the batch effect removed?

Thank you for your constructive suggestions. To correct for batch effects across datasets, we used the Harmony package. The integration results are presented in Figure 1B.

2. What is the marker gene set for CAFs? Where was it obtained from?

Thank you for your suggestion. We used ACTA2, TAGLN, and CTHRC1 as marker genes for cancer-associated fibroblasts (CAFs), and DCN, LUM, and COL1A1 as markers for normal fibroblasts. The marker genes for CAFs were obtained from the following studies (PMID: 37463917, 33622705, and 32434947), which have been cited in the main text.

3. Why were only two patients selected for the immunofluorescence staining experiment? Are there specific values for spatial distance to illustrate the research conclusions? It is recommended to use HALO spatial coordinate analysis software to define the distance between different cell types.

Thank you for your constructive suggestions. At present, the number of samples containing both tumor tissue and adjacent normal regions is limited. We plan to collect additional samples in future studies to further validate this observation. In response to your suggestion, we performed HALO spatial coordinate analysis, which revealed that apCAFs and M2 macrophages are spatially proximal at the tumor margin. The results of this analysis have been included in the supplementary materials (Newly Added Supplementary Figure 3).

4. How is the spatial co-localization of AQP4 and M2 macrophage markers in the same area in ST defined? Was the spatial co-localization score used?

Thank you for your suggestion. While our initial assessment of AQP4 and M2 macrophage co-localization was based on overlapping expression patterns, we have now incorporated a quantitative spatial co-localization score analysis, as recommended. These new results, obtained through normalized expression products and kNN-based neighborhood analysis, further support our original conclusion and have been included in Supplementary Figure 3.

5. What is the expression pattern of AQP4 in immunofluorescence staining?

Thank you for your suggestion. Immunofluorescence results show that the gene AQP4 is primarily expressed in apCAFs.

Reviewer #2: Ren et al. analyzed publicly available scRNA-seq and bulk RNA sequencing datasets, as well as ST data from glioma patients. They investigated nine distinct CAF subtypes and mostly focused on apCAFs. Their data shows that apCAF2 is co-localized with M2 macrophages and there is a crosstalk between them via the TGF-b pathway. Patients with high apCAFs scores exhibited stronger immune resistance than patients with low apCAFs scores. Furthermore, they found that AQP4 is a specific marker for apCAFs and high expression of AQP4 in the tumor microenvironment indicated the immune resistance as well. Overall, the manuscript is well-written and presents interesting data.

We are truly grateful for your thoughtful and constructive review of our manuscript. We greatly appreciate your insightful comments and your positive evaluation of our study.

Attachment Submitted filename: Response to Reviewers.docx

# Editor report: Decision Letter 1

**DOI:** 10.1371/journal.pone.0329801.r003

**Authors:** Zeyneb Kurt

22 Jul 2025

A multi-omics atlas of CAF subtypes reveals apCAF–M2 macrophage interactions driving immune resistance in glioma

PONE-D-25-29408R1

Dear Dr. Sun,

We’re pleased to inform you that your manuscript has been judged scientifically suitable for publication and will be formally accepted for publication once it meets all outstanding technical requirements.

Within one week, you’ll receive an e-mail detailing the required amendments. When these have been addressed, you’ll receive a formal acceptance letter and your manuscript will be scheduled for publication.

An invoice will be generated when your article is formally accepted. Please note, if your institution has a publishing partnership with PLOS and your article meets the relevant criteria, all or part of your publication costs will be covered. Please make sure your user information is up-to-date by logging into Editorial Manager at Editorial Manager® and clicking the ‘Update My Information' link at the top of the page. If you have any questions relating to publication charges, please contact our Author Billing department directly at authorbilling@plos.org.

If your institution or institutions have a press office, please notify them about your upcoming paper to help maximize its impact. If they’ll be preparing press materials, please inform our press team as soon as possible -- no later than 48 hours after receiving the formal acceptance. Your manuscript will remain under strict press embargo until 2 pm Eastern Time on the date of publication. For more information, please contact onepress@plos.org.

Kind regards,

Zeyneb Kurt, PhD

Academic Editor

PLOS ONE

Additional Editor Comments (optional):

Reviewers' comments:

# Editor report: Acceptance letter

**DOI:** 10.1371/journal.pone.0329801.r004

**Authors:** Zeyneb Kurt

PONE-D-25-29408R1

PLOS ONE

Dear Dr. Sun,

I'm pleased to inform you that your manuscript has been deemed suitable for publication in PLOS ONE. Congratulations! Your manuscript is now being handed over to our production team.

At this stage, our production department will prepare your paper for publication. This includes ensuring the following:

* All references, tables, and figures are properly cited

* All relevant supporting information is included in the manuscript submission,

* There are no issues that prevent the paper from being properly typeset

You will receive further instructions from the production team, including instructions on how to review your proof when it is ready. Please keep in mind that we are working through a large volume of accepted articles, so please give us a few days to review your paper and let you know the next and final steps.

Lastly, if your institution or institutions have a press office, please let them know about your upcoming paper now to help maximize its impact. If they'll be preparing press materials, please inform our press team within the next 48 hours. Your manuscript will remain under strict press embargo until 2 pm Eastern Time on the date of publication. For more information, please contact onepress@plos.org.

If we can help with anything else, please email us at customercare@plos.org.

Thank you for submitting your work to PLOS ONE and supporting open access.

Kind regards,

PLOS ONE Editorial Office Staff

on behalf of

Dr. Zeyneb Kurt

Academic Editor

PLOS ONE
